# Supplementary material for: Importance of interface open circuit potential on aqueous hydrogenolytic reduction of benzyl alcohol over Pd/C
Source: Nat Commun. 2022 Dec 27;13:7967. doi: 10.1038/s41467-022-35554-1 (PMC9794693; doi:10.1038/s41467-022-35554-1)
Supplement: Supplementary file 1 — Supplementary Information [file 41467_2022_35554_MOESM1_ESM.pdf]

Importance of Interface Open Circuit Potential on Aqueous Hydrogenolytic Reduction of Benzyl Alcohol over Pd/C

Guanhua Cheng[1,2], Wei Zhang[1,3], Andreas Jentys[1], Erika E. Ember [1], Oliver Y. Gutiérrez[4], Yue Liu[1,3]*, and Johannes A. Lercher[1,4]*

[1]Technische Universität München, Department of Chemistry and Catalysis Research Center, Lichtenbergstraβe 4, Garching, D-85748, Germany
[2]Key Laboratory for Liquid-Solid Structural Evolution and Processing of Materials (Ministry of Education), School of Materials Science and Engineering, Shandong University, Jingshi Road 17923, Jinan 250061, PR China
[3]Shanghai Key Laboratory of Green Chemistry and Chemical Processes, School of Chemistry and Molecular Engineering, East China Normal University, Shanghai, PR China
[4]Institute for Integrated Catalysis, Pacific Northwest National Laboratory, 902 Battelle Boulevard, Richland, WA 99352, USA

Supplementary Information

Supplementary Tables

Table S1. Textural properties of the Pd supported catalysts.

Catalyst	Metal loading (wt.%)	BET surface area ($m^2 \cdot g^{-1}$)	Particle size (nm)[a]	Dispersion (%)[b]
Pd/C	5	1034	2.9	33

[a] measured by TEM.
[b] measured by hydrogen chemisorption.

Supplementary Figures

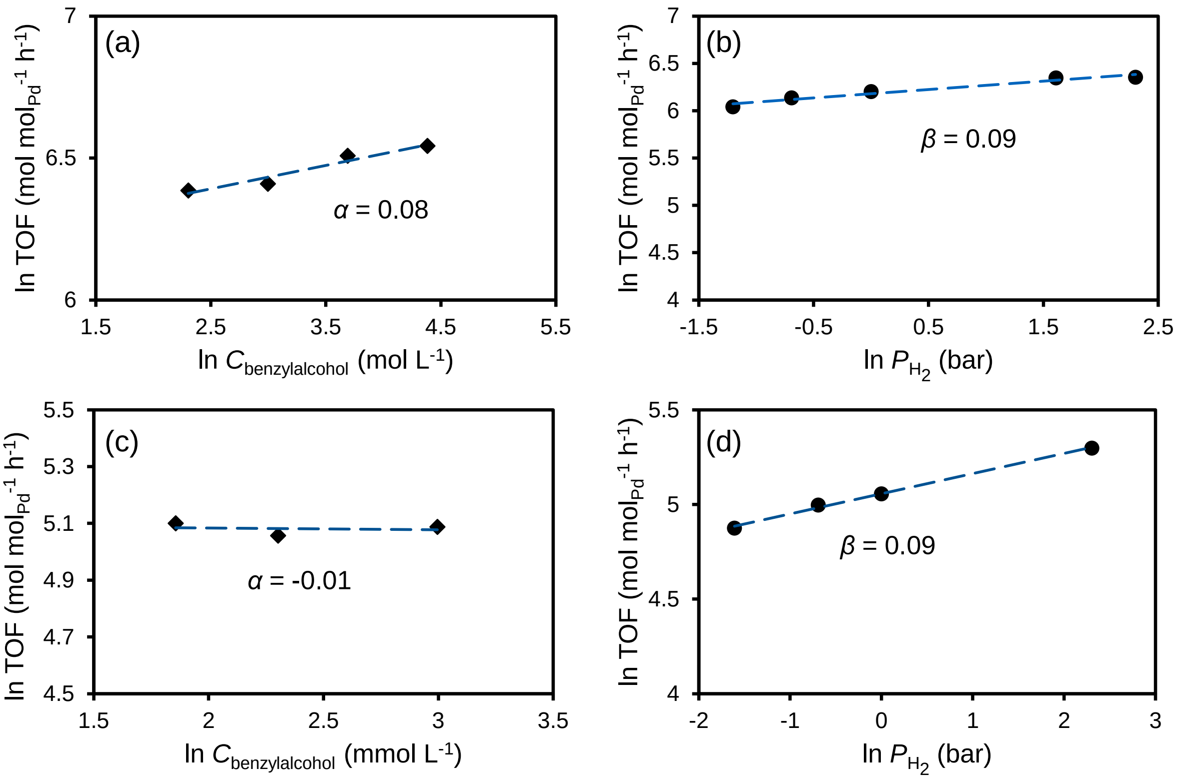

Figure S1 | Reaction orders in benzyl alcohol and hydrogen. (a) TOF as a function of benzyl alcohol concentration (6.4-20 mM) at 1 bar H_2 and (b) TOF as a function of hydrogen pressure (0.3-1bar) with the benzyl alcohol concentration of 10 mM in 0.2 M phosphate buffer (pH 2.5). (c) TOF as a function of benzyl alcohol concentration (10-32 mM) at 1 bar H_2 and (d) TOF as a function of hydrogen pressure (0.3-1bar) with the benzyl alcohol concentration of 10 mM in 0.2 M acetate buffer (pH 5). α and β are the apparent reaction orders with respect to benzaldehyde and H_2.

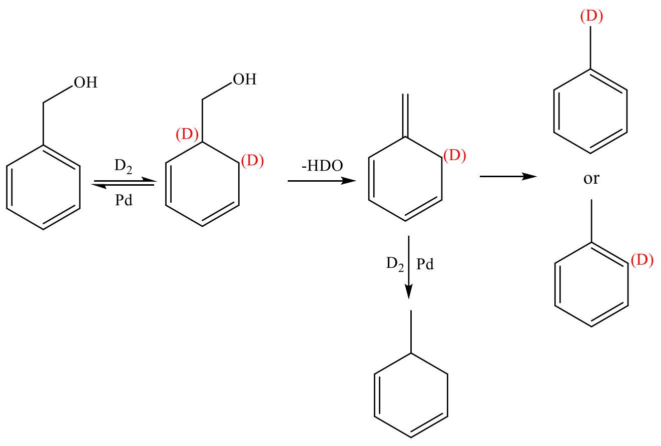

Figure S2 | Reaction mechanism of benzyl alcohol reductive elimination through hydrogenation-dehydration-hydrogen scrambling.

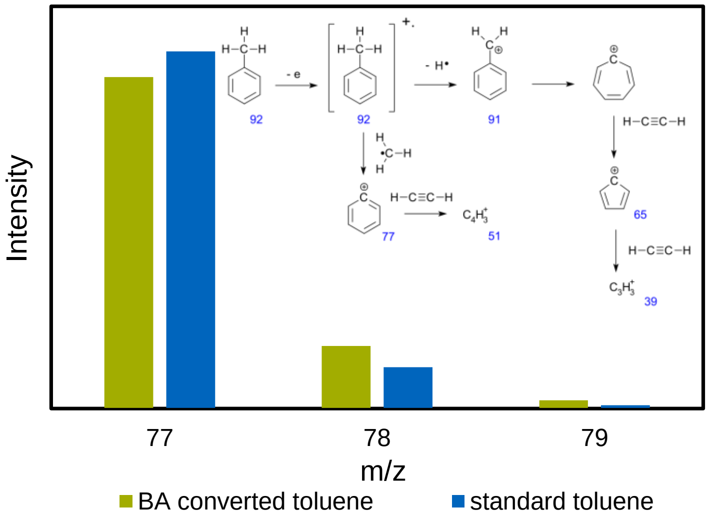

Figure S3 | Mass spectra of fragment ions of toluene produced in the reaction of benzyl alcohol with D_2 and standard toluene. Reaction condition: 0.2 M deuterated buffer solution, 298 K and 1 bar D_2.

Supplementary Notes

Note S1. Deuterium study

In order to check if the benzyl alcohol reacts via the pathway of partial hydrogenation - dehydration – re-hydrogenation (Figure S2), D_2 and D_2O were used instead of H_2 and H_2O, respectively. If the reaction follows this pathway, the produced toluene product should have D-labeled at aromatic ring because the pathway involves hydrogenation of the aromatic ring as an elementary step (Figure S2). However, after comparing mass spectra of the toluene produced in the reaction and standard toluene, it is concluded that no deuterium was labeled on the aromatic ring (Figure S3). Therefore, the partial hydrogenation - dehydration – re-hydrogenation pathway is excluded.

Note S2 Derivation of the rate equations

The sum of the fractional coverages for species *i* (θ_i), which include the adsorbed H adatom (H_{ad}), adsorbed benzyl alcohol (BA_{ad}), protonated benzyl alcohol ($BAH^+{}_{ad}$), together with that of the unoccupied site (*), equals unity:

$$\theta* + \theta_{H_{ad}} + \theta_{BA_{ad}} + \theta_{BAH^+{}_{ad}} = 1 \qquad (1)$$

According to the elementary steps, the relations can be obtained as below,

$$BA(aq.) \rightleftharpoons BA_{ad} \qquad \text{(Step a)}$$

$$BA_{ad} + H^+(aq.) \rightleftharpoons BAH^+{}_{ad} \qquad \text{(Step b)}$$

$$BAH^+{}_{ad} \rightleftharpoons TS \xrightarrow{k_BT/h} C_6H_5CH_2{}^+{}_{ad} \qquad \text{(Step c)}$$

Therefore, based on the equilibrium, the chemical potential of the species above shows the relation as following,

$$\mu_{BAaq} = \mu_{BA_{ad}} \qquad (2)$$

$$\mu_{BA_{ad}} + \mu_{H^+} = \mu_{BAH^+{}_{ad}} = \mu_{TS} \qquad (3)$$

$$\mu_{H_2} = 2\mu_{H_{ad}} \qquad (4)$$

In which μ_{BAaq}, μ_{BAad}, μ_{H_2}, μ_{Had}, μ_{H+}, μ_{BAH^+ad} and μ_{TS} are chemical potential of benzyl alcohol in bulk, sorbed benzyl alcohol, gas H_2, sorbed H, hydronium ion in bulk, sorbed protonated benzyl alcohol and transition state, respectively. The expressions for each chemical potential are

$$\mu_{BA_{aq}} = \mu^o_{BA_{aq}} + RT\ln a_{BA_{aq}} \qquad (5)$$

$$\mu_{H_{ad}} = \mu^o_{H_{ad}} + RT\ln\frac{\theta_{H_{ad}}}{\theta*} \qquad (6a)$$

$$\frac{\theta_{H_{ad}}}{\theta*} = exp\left(\frac{\mu_{H_{ad}} - \mu^o_{H_{ad}}}{RT}\right) \qquad (6b)$$

$$\mu_{H_2} = \mu^o_{H_2} + RT\ln P_{H_2} \qquad (7)$$

$$\mu_{BA_{ad}} = \mu^o_{BA_{ad}} + RT\ln\frac{\theta_{BA_{ad}}}{\theta*} \qquad (8a)$$

$$\frac{\theta_{BA_{ad}}}{\theta*} = exp\left(\frac{\mu_{BA_{ad}} - \mu^o_{BA_{ad}}}{RT}\right) \qquad (8b)$$

$$\mu_{BAH^+{}_{ad}} = \mu^o_{BAH^+{}_{ad}} + RT\ln\frac{\theta_{BAH^+{}_{ad}}}{\theta*} + F\varphi_{BAH^+{}_{ad}} \qquad (9a)$$

$$\frac{\theta_{\mathrm{BAH^+}_{\mathrm{ad}}}}{\theta*} = exp\left(\frac{\mu_{\mathrm{BAH^+}_{\mathrm{ad}}} - \mu^{\circ}_{\mathrm{BAH^+}_{\mathrm{ad}}} - F\varphi_{\mathrm{BAH^+}_{\mathrm{ad}}}}{RT}\right) \quad (9b)$$

$$\mu_{\mathrm{H^+}} = \mu^{\circ}_{\mathrm{H^+}} + RT\ln a_{\mathrm{H^+}} + F\varphi_{\mathrm{B}} \quad (10)$$

$$\mu_{\mathrm{TS}} = \mu^{\circ}_{\mathrm{TS}} + RT\ln\frac{\theta_{\mathrm{TS}}}{\theta*} + F\varphi_{\mathrm{TS}} \quad (11a)$$

$$\frac{\theta_{\mathrm{TS}}}{\theta*} = exp\left(\frac{\mu_{\mathrm{TS}} - \mu^{\circ}_{\mathrm{TS}} - F\varphi_{\mathrm{TS}}}{RT}\right) \quad (11b)$$

in which $\varphi_{\mathrm{BAH^+}_{\mathrm{ad}}}$, φ_{B} and φ_{TS} are the electrochemical potential at the position of $\mathrm{BAH^+}_{\mathrm{ad}}$, bulk phase and transition state. Generally, φ_{B} is denoted as 0. Substitute Equation 1, 6b, 8b and 9b in $1/\theta*$, we can obtain

$$\frac{1}{\theta*} = \frac{\theta* + \theta_{\mathrm{H_{ad}}} + \theta_{\mathrm{BA_{ad}}} + \theta_{\mathrm{BAH^+}_{\mathrm{ad}}}}{\theta*}$$

$$= 1 + exp\left(\frac{\mu_{\mathrm{H_{ad}}} - \mu^{\circ}_{\mathrm{H_{ad}}}}{RT}\right) + exp\left(\frac{\mu_{\mathrm{BA_{ad}}} - \mu^{\circ}_{\mathrm{BA_{ad}}}}{RT}\right) + exp\left(\frac{\mu_{\mathrm{BAH^+}_{\mathrm{ad}}} - \mu^{\circ}_{\mathrm{BAH^+}_{\mathrm{ad}}} - F\varphi_{\mathrm{BAH^+}_{\mathrm{ad}}}}{RT}\right)$$

(12)

Then substitute Equation 12 into Equation 11b, after arrangement, we can obtain the expression of θ_{TS},

$$\theta_{\mathrm{TS}} = \theta* \cdot exp\left(\frac{\mu_{\mathrm{TS}} - \mu^{\circ}_{\mathrm{TS}} - F\varphi_{\mathrm{TS}}}{RT}\right)$$

$$= \frac{exp\left(\frac{\mu_{\mathrm{TS}} - \mu^{\circ}_{\mathrm{TS}} - F\varphi_{\mathrm{TS}}}{RT}\right)}{1 + exp\left(\frac{\mu_{\mathrm{H_{ad}}} - \mu^{\circ}_{\mathrm{H_{ad}}}}{RT}\right) + exp\left(\frac{\mu_{\mathrm{BA_{ad}}} - \mu^{\circ}_{\mathrm{BA_{ad}}}}{RT}\right) + exp\left(\frac{\mu_{\mathrm{BAH^+}_{\mathrm{ad}}} - \mu^{\circ}_{\mathrm{BAH^+}_{\mathrm{ad}}} - F\varphi_{\mathrm{BAH^+}_{\mathrm{ad}}}}{RT}\right)} \quad (13)$$

Therefore, the rate equation is obtained as follows,

$$r = \frac{k_B T}{h} \cdot \theta_{\mathrm{TS}}$$

$$= \frac{k_B T}{h} \cdot \frac{exp\left(\frac{\mu_{\mathrm{TS}} - \mu^{\circ}_{\mathrm{TS}} - F\varphi_{\mathrm{TS}}}{RT}\right)}{1 + exp\left(\frac{\mu_{\mathrm{H_{ad}}} - \mu^{\circ}_{\mathrm{H_{ad}}}}{RT}\right) + exp\left(\frac{\mu_{\mathrm{BA_{ad}}} - \mu^{\circ}_{\mathrm{BA_{ad}}}}{RT}\right) + exp\left(\frac{\mu_{\mathrm{BAH^+}_{\mathrm{ad}}} - \mu^{\circ}_{\mathrm{BAH^+}_{\mathrm{ad}}} - F\varphi_{\mathrm{BAH^+}_{\mathrm{ad}}}}{RT}\right)} \quad (14a)$$

$$r = \frac{k_B T}{h} \cdot \frac{exp\left(\frac{\mu_{\mathrm{BA_{ad}}} + \mu_{\mathrm{H^+}} - \mu^{\circ}_{\mathrm{TS}} - F\varphi_{\mathrm{TS}}}{RT}\right)}{1 + exp\left(\frac{1/2\mu_{\mathrm{H_2}} - \mu^{\circ}_{\mathrm{H_{ad}}}}{RT}\right) + exp\left(\frac{\mu_{\mathrm{BA_{ad}}} - \mu^{\circ}_{\mathrm{BA_{ad}}}}{RT}\right) + exp\left(\frac{\mu_{\mathrm{BA_{ad}}} + \mu_{\mathrm{H^+}} - \mu^{\circ}_{\mathrm{BAH^+}_{\mathrm{ad}}} - F\varphi_{\mathrm{BAH^+}_{\mathrm{ad}}}}{RT}\right)}$$

$$= \frac{k_B T}{h} \cdot \frac{exp\left(\frac{\mu_{\mathrm{BA_{aq}}} + \mu_{\mathrm{H^+}} - \mu^{\circ}_{\mathrm{TS}} - F\varphi_{\mathrm{TS}}}{RT}\right)}{1 + exp\left(\frac{1/2\mu_{\mathrm{H_2}} - \mu^{\circ}_{\mathrm{H_{ad}}}}{RT}\right) + exp\left(\frac{\mu_{\mathrm{BA_{aq}}} - \mu^{\circ}_{\mathrm{BA_{ad}}}}{RT}\right) + exp\left(\frac{\mu_{\mathrm{BA_{aq}}} + \mu_{\mathrm{H^+}} - \mu^{\circ}_{\mathrm{BAH^+}_{\mathrm{ad}}} - F\varphi_{\mathrm{BAH^+}_{\mathrm{ad}}}}{RT}\right)} \quad (14b)$$

Then with the rate equation, and partial derivative of ln$r$ with respect to lnC_{BAaq}, lnP_{H2}, ln$a_{\mathrm{H+}}$ and φ_{M}, we can obtain

$$\frac{\partial lnr}{\partial lna_{\mathrm{BA_{aq}}}}=\frac{\partial lnr}{\partial\left(\frac{\mu_{\mathrm{BA_{aq}}}-\mu^{\mathrm{o}}_{\mathrm{BA_{aq}}}}{RT}\right)}=RT\frac{\partial lnr}{\partial\mu_{\mathrm{BA_{aq}}}}$$
$$=\frac{\partial(\mu_{\mathrm{BA_{ad}}}+\mu_{\mathrm{H^+}}-F\varphi_{\mathrm{TS}})}{\partial\mu_{\mathrm{BA_{aq}}}}-\theta_{\mathrm{BA_{ad}}}\frac{\partial\mu_{\mathrm{BA_{aq}}}}{\partial\mu_{\mathrm{BA_{aq}}}}-\theta_{\mathrm{BAH^+{}_{ad}}}\frac{\partial(\mu_{\mathrm{BA_{aq}}}+\mu_{\mathrm{H^+}}-F\varphi_{\mathrm{BAH^+{}_{ad}}})}{\partial\mu_{\mathrm{BA_{aq}}}} \quad (15)$$
$$=1-\theta_{\mathrm{BA_{ad}}}-\theta_{\mathrm{BAH^+{}_{ad}}}$$

$$\frac{\partial lnr}{\partial lnP_{\mathrm{H_2}}}=\frac{\partial lnr}{\partial\left(\frac{\mu_{\mathrm{H_2}}-\mu^{\mathrm{o}}_{\mathrm{H_2}}}{RT}\right)}=RT\frac{\partial lnr}{\partial\mu_{\mathrm{H_2}}}$$
$$=\frac{\partial(\mu_{\mathrm{BA_{aq}}}+\mu_{\mathrm{H^+}}-F\varphi_{\mathrm{TS}})}{\partial\mu_{\mathrm{H_2}}}-\frac{1}{2}\theta_{\mathrm{H_{ad}}}\frac{\partial\mu_{\mathrm{H_2}}}{\partial\mu_{\mathrm{H_2}}}-\theta_{\mathrm{BA_{ad}}}\frac{\partial\mu_{\mathrm{BA_{ad}}}}{\partial\mu_{\mathrm{H_2}}}-\theta_{\mathrm{BAH^+{}_{ad}}}\frac{\partial(\mu_{\mathrm{BA_{aq}}}+\mu_{\mathrm{H^+}}-F\varphi_{\mathrm{BAH^+{}_{ad}}})}{\partial\mu_{\mathrm{H_2}}}$$
$$=-\frac{1}{2}\theta_{\mathrm{H_{ad}}}-F\frac{\partial\varphi_{\mathrm{TS}}}{\partial\mu_{\mathrm{H_2}}}+\theta_{\mathrm{BAH^+{}_{ad}}}F\frac{\partial\varphi_{\mathrm{BAH^+{}_{ad}}}}{\partial\mu_{\mathrm{H_2}}}$$

(16)

$$\frac{\partial lnr}{\partial lna_{\mathrm{H^+}}}=\frac{\partial lnr}{\partial\left(\frac{\mu_{\mathrm{H^+}}-\mu^{\mathrm{o}}_{\mathrm{H^+}}}{RT}\right)}$$
$$=\frac{\partial(\mu_{\mathrm{BA_{aq}}}+\mu_{\mathrm{H^+}}-F\varphi_{\mathrm{TS}})}{\partial\mu_{\mathrm{H^+}}}-\frac{1}{2}\theta_{\mathrm{H_{ad}}}\frac{\partial\mu_{\mathrm{H_2}}}{\partial\mu_{\mathrm{H^+}}}-\theta_{\mathrm{BA_{ad}}}\frac{\partial\mu_{\mathrm{BA_{aq}}}}{\partial\mu_{\mathrm{H^+}}}-\theta_{\mathrm{BAH^+{}_{ad}}}\frac{\partial(\mu_{\mathrm{BA_{aq}}}+\mu_{\mathrm{H^+}}-F\varphi_{\mathrm{BAH^+{}_{ad}}})}{\partial\mu_{\mathrm{H^+}}}$$
$$=1-\theta_{\mathrm{BAH^+{}_{ad}}}-F\frac{\partial\varphi_{\mathrm{TS}}}{\partial\mu_{\mathrm{H^+}}}+\theta_{\mathrm{BAH^+{}_{ad}}}F\frac{\partial\varphi_{\mathrm{BAH^+{}_{ad}}}}{\partial\mu_{\mathrm{H^+}}}$$

(17)

$$\frac{\partial lnr}{\partial\varphi_{\mathrm{M}}}=\frac{1}{RT}\left[\frac{\partial(\mu_{\mathrm{BA_{aq}}}+\mu_{\mathrm{H^+}}-F\varphi_{\mathrm{TS}})}{\partial\varphi_{\mathrm{M}}}-\frac{1}{2}\theta_{\mathrm{H_{ad}}}\frac{\partial\mu_{\mathrm{H_2}}}{\partial\varphi_{\mathrm{M}}}-\theta_{\mathrm{BA_{ad}}}\frac{\partial\mu_{\mathrm{BA_{ad}}}}{\partial\varphi_{\mathrm{M}}}-\theta_{\mathrm{BAH^+{}_{ad}}}\frac{\partial(\mu_{\mathrm{BA_{aq}}}+\mu_{\mathrm{H^+}}-F\varphi_{\mathrm{BAH^+{}_{ad}}})}{\partial\varphi_{\mathrm{M}}}\right]$$
$$=\frac{F}{RT}\left(-\frac{\partial\varphi_{\mathrm{TS}}}{\partial\varphi_{\mathrm{M}}}+\theta_{\mathrm{BAH^+{}_{ad}}}\frac{\partial\varphi_{\mathrm{BAH^+{}_{ad}}}}{\partial\varphi_{\mathrm{M}}}\right)$$

(18)

Due to the equilibrium of hydronium ions and gas hydrogen, the electrode potential of Pd can be written as,

$$\varphi_{\mathrm{M}}=\varphi_{\mathrm{SHE}}+\frac{RT}{F}ln\frac{a_{\mathrm{H^+}}}{\sqrt{P_{\mathrm{H_2}}}} \quad (19)$$

In which φ_{SHE} is standard hydrogen electrode (SHE), which is often used as a reference and generally defined it as 0 V for convenience. It should be noted that φ_{M} is contact potential, and the electron work function of Pd is not considered here. With Equation 19, we can obtain,

$$\frac{\partial \varphi_{\mathrm{M}}}{\partial lnP_{\mathrm{H_2}}} = -\frac{RT}{2F} \quad (20a)$$

$$\frac{\partial \varphi_{\mathrm{M}}}{\partial \mu_{\mathrm{H_2}}} = -\frac{1}{2F} \quad (20b)$$

$$-F = \frac{\partial \mu_{\mathrm{H_2}}}{2\partial \varphi_{\mathrm{M}}} \quad (20c)$$

$$\frac{\partial \varphi_{\mathrm{M}}}{\partial lna_{\mathrm{H^+}}} = \frac{RT}{F} \quad (21a)$$

$$\frac{\partial \varphi_{\mathrm{M}}}{\partial \mu_{\mathrm{H^+}}} = \frac{1}{F} \quad (21b)$$

$$F = \frac{\partial \mu_{\mathrm{H^+}}}{\partial \varphi_{\mathrm{M}}} \quad (21c)$$

Substitute Equation 20c into Equation 16,

$$\frac{\partial lnr}{\partial lnP_{\mathrm{H_2}}} = -\frac{1}{2}\theta_{\mathrm{H_{ad}}} + \frac{\partial \varphi_{\mathrm{TS}}}{2\partial \varphi_{\mathrm{M}}} - \theta_{\mathrm{BAH^+_{ad}}} \frac{\partial \varphi_{\mathrm{BAH^+_{ad}}}}{2\partial \varphi_{\mathrm{M}}} \quad (22)$$

Substitute Equation 21c into Equation 17,

$$\frac{\partial lnr}{\partial lna_{\mathrm{H^+}}} = 1 - \theta_{\mathrm{BAH^+_{ad}}} - \frac{\partial \varphi_{\mathrm{TS}}}{\partial \varphi_{\mathrm{M}}} + \theta_{\mathrm{BAH^+_{ad}}} \frac{\partial \varphi_{\mathrm{BAH^+_{ad}}}}{\partial \varphi_{\mathrm{M}}} \quad (23)$$

Equation 14a, 14b, 15, 22, 23, 18 are the equation 13a, 13b, 14, 15,16, 17 in the main text, respectively.
